# Development of a food allergy educative program: in-person vs online

**DOI:** 10.1186/2045-7022-3-S3-P136

**Published:** 2013-07-25

**Authors:** J Contreras-Porta, A Ruiz-Baqués, F Capel, M Ariño, A Zorrozua

**Affiliations:** 1 La Paz University Hospital, Madrid, Spain; 2 Autonomous University of Barcelona, Topping Medical Research, Barcelona, Spain; 3 AEPNAA, Spanish Association of Food and Latex Allergy, Madrid, Spain; 4 Immunitas Vera, Catalonian Association of Food and Latex Allergy, Barcelona, Spain; 5 Elikalte, Basque Association of Food Allergy, Bilbao, Spain

## Background

Anaphylaxis is a rapid onset serious allergic reaction that may cause death. Food allergy is the most frequent cause of anaphylactic reactions and it is estimated to affect 7% of children in Spain. However, there is a lack of educational programsfor families in this condition.The growing use of social media and Participatory Medicine (PM) is allowing patients to take greater control of their own healthcare.

The aim was to create an educational program that includes resources forin-person workshops and an online self-care education program for parents of children aged <12 years old who are at high risk of anaphylaxis due tofood allergy.

## Methods

This project has been conceived, developed and carried out under the PM model. Everything has been agreed upon by the principal Spanish patient associations on food allergy (AEPNAA, Immunitas Vera and Elikalte), and by health professionals and researchers. It received the approval of the Ethics Committee of the University Hospital La Paz, Madrid, and of the main scientific Spanish allergy societies SEAIC, SEICAP and AEP.

Recruitment of participants was carried out via patient associations and the website http://www.alergiayalimentos.com. Those who accepted were randomly assigned to either an in-person or online educative program. Of a total of 300 participant families, 150 are attending in-person educational programs carried out in eight Spanish cities and led by health professionals and expert patients; the other 150 families are following the e-Learning platform.

The project started in November 2012. Satisfaction levels and increases in knowledge are being assessed through an *adhoc* questionnaire administered before and after the educational program.

## Results

As of today, 300 families with children at high risk of anaphylaxis due to food allergy have agreed to participate. Participating families’ satisfaction levels, as well as the degree of knowledge before and after the educational program, will be assessed and compared in both groups (in-person and online).

Several media types were created, including educational videos, games, storytelling in order to improve knowledge of food allergy, how to avoid allergens, treat allergic reactions and how to use theEpinephrine Auto-Injector.

## Conclusion

CESA project has been well accepted by families of children with severe food allergies and is likely to show the need for health education in order to support proper management of the condition, thereby achieving a higher quality of life.

## Disclosure of interest

None declared.

